# Sinusitis and Late-Onset Asthma: A Red Flag of Eosinophilic Granulomatosis With Polyangiitis

**DOI:** 10.7759/cureus.35512

**Published:** 2023-02-27

**Authors:** Lídia Gomes, Sandra D Santos, Samuel Pereira, João Rua, Jorge Fortuna

**Affiliations:** 1 Pulmonology, University Hospital Center of Coimbra, Coimbra, PRT; 2 Internal Medicine, University Hospital Center of Coimbra, Coimbra, PRT; 3 Intensive Care Unit, University Hospital Center of Coimbra, Coimbra, PRT

**Keywords:** vasculitis, eosinophilia, sinusitis, asthma, eosinophilic granulomatosis with polyangiitis

## Abstract

Eosinophilic granulomatosis with polyangiitis (EGPA) is a rare systemic disease that develops with necrotizing granulomatous inflammation and is characterized by eosinophilia, asthma, and small vessel vasculitis. We report the case of a 74-year-old woman with a history of asthma, admitted to the Emergency Room with fever, headache, general malaise, weight loss and night sweats with one-month evolution, previously medicated with antibiotics without improvement. She presented with sinus palpation tenderness and lower leg bilateral sensitivity impairment. Laboratory tests showed neutrophilia and eosinophilia, normocytic anemia and elevated erythrocyte sedimentation rate and C-reactive protein. A computed tomography revealed sphenoid and maxillary sinusitis. Blood cultures and lumbar puncture were innocent. An extended autoimmune panel exposed a strong positive perinuclear anti-neutrophil cytoplasmic antibody - myeloperoxidase (pANCA-MPO). Sinus biopsy showed tissue infiltration by eosinophils, confirming EGPA. Corticosteroid (1 mg/kg/day) treatment was started with gradual improvement. Six months later there were no signs of active disease under prednisolone 10 mg and azathioprine 50 mg/day. This case highlights that refractory sinusitis in the presence of constitutional syndrome and peripheral eosinophilia should alert clinicians to the possibility of EGPA, particularly in patients with late-onset asthma.

## Introduction

Eosinophilic granulomatosis with polyangiitis (EGPA), previously known as Churg-Strauss syndrome, is a rare systemic anti-neutrophil cytoplasmic antibody (ANCA)-associated necrotizing granulomatous vasculitis that affects small and medium size vessels, usually characterized by blood and tissue eosinophilia and history of asthma [1]. It is a rare pathology with an equal incidence between genders and can develop at any age, but typically appears at the sixth decade of life [2,3].

EGPA is characterized by three stages: the prodromic phase occurs usually with late onset of asthma and rhinitis; the eosinophilic phase follows with peripheral and tissue eosinophilia in several organs; lastly, the vasculitic phase is characterized by systemic vasculitis affecting peripheral nerves, heart, lung, gastrointestinal tract, and kidney [4]. The severity and prognosis of the disease vary, depending on the disease stage. The diagnosis requires a high level of suspicion, specially before the vasculitic phase, when most organ damage occurs [5].

We highlight a case where the association of refractory sinusitis and late-onset asthma in an elderly woman, pointed to the diagnosis and allowed treatment in the initial stages.

This article was previously presented as a poster at the 7^th^ Pneumo Update Europe, virtual event, October 30-31, 2020.

## Case presentation

A 74-year-old woman was admitted to the Emergency Room (ER) with persistent complaints of fever, headache, general malaise, weight loss and night sweats during the previous month. She also referred abnormal sensitivity in the lower limbs, dry cough, frontal headache and mandibular claudication. In the previous month, she had been medicated with levofloxacin and cefixime for a supposed urinary tract infection and sinusitis, without improvement. She had a history of essential hypertension and difficult-to-control asthma diagnosed 11 years before. There were no smoking/alcoholic habits or other exposures. Chronic medication consisted of budesonide/formoterol 160/4.5 ug inhaler, montelukast 10 mg, lisinopril 10 mg and rosuvastatin 10 mg. She was allergic to doxycycline.

Upon physical examination, the patient was subfebrile, hemodynamically stable, and eupneic without oxygen. She had hydrated but pallid skin and was acyanotic and anicteric but had a weakened appearance. Her cognition was normal. Non-palpable thyroid and only a small right supraclavicular adenopathy were found. Cardiac, pulmonary, and abdominal examinations were unremarkable. There were no skin lesions, evidence of lower limbs edema or signs of venous thromboembolism. She had pain from palpation of the sinuses and impaired sensation in the lower half of the legs, with normal reflexes and proprioception; the remaining neurological examination was normal.

Analysis showed normocytic anemia (hemoglobin 9.8 g/dL), leukocytosis (17.57 x 10^3^/uL) with neutrophilia (56.6%) and eosinophilia (26.5%), thrombocytosis (platelets 816 x 10^3^/uL) and very high erythrocyte sedimentation rate (ESR) 96 mm/h (N < 35) and C-reactive protein (CRP) 17.9 mg/dL (N < 0.8); peripheral blood smear showed eosinophilia and thrombocytosis, without other remarkable features. International normalized ratio (INR) was 1.24; alkaline phosphatase (ALP) 195 U/L (N: 38-126), gamma-glutamyl transferase (GGT) 183 U/L (N: 12-58), aspartate aminotransferase (AST) 51 U/L (N: <35), alanine aminotransferase (ALT) 48 U/L (N: <34), lactate dehydrogenase (LDH) 229 U/L (N: 125-220); renal function and blood ion levels were normal. Blood cultures, urine and cerebrospinal fluid (CSF) analysis revealed no abnormalities. Chest radiograph was normal.

**Figure 1:**
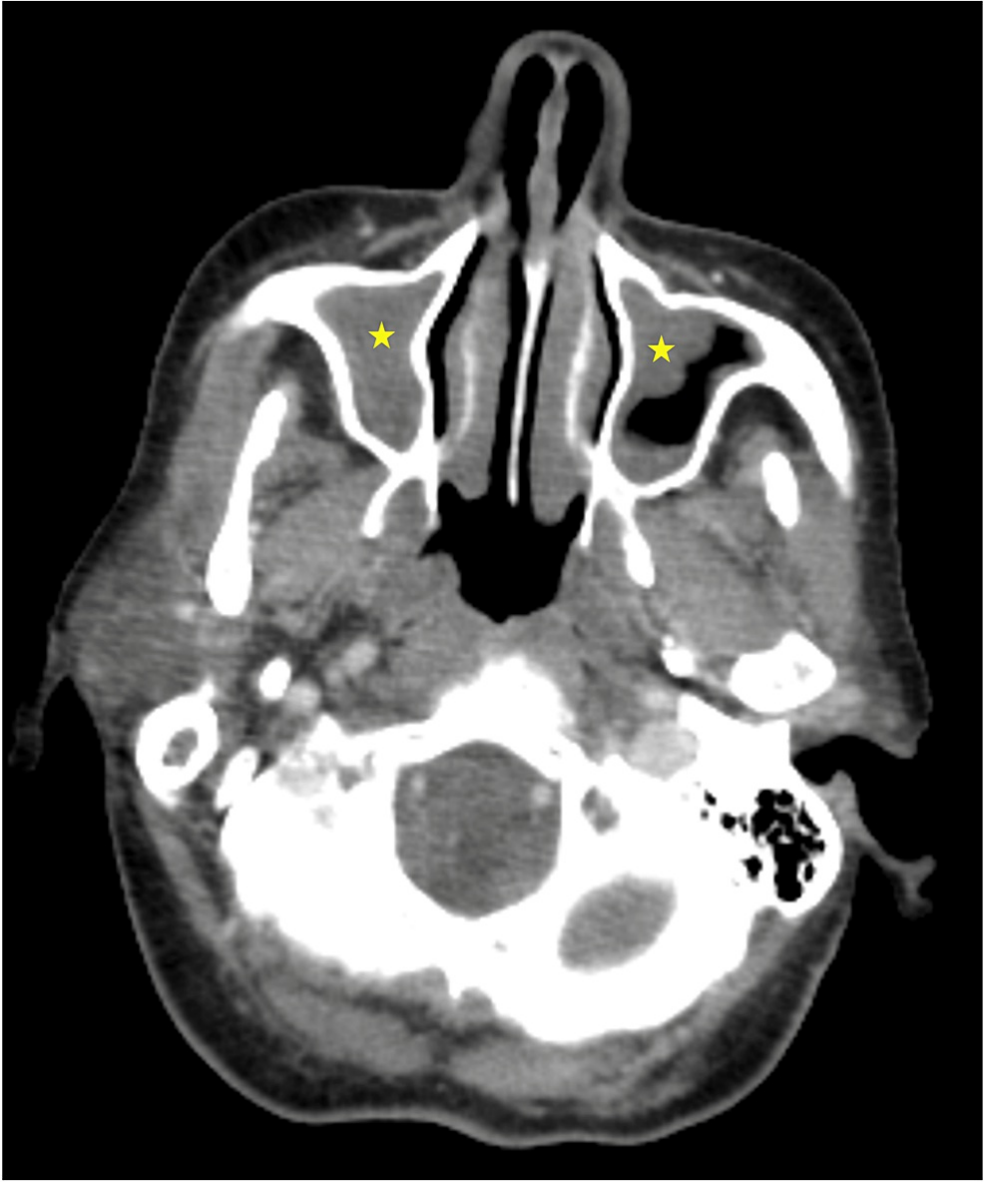
Maxillary computed tomography (CT) scan showing acute inflammatory changes in the maxillary sinus (stars).

A maxillofacial computed tomography (CT) showed sphenoid and maxillary sinusitis (Figure 1) and ceftriaxone was prescribed by the otorhinolaryngologist. The patient was admitted for monitoring and further investigation.

Further study showed serum protein electrophoresis had an alpha-1 and alpha-2 peak, normal immunoglobulins, except for IgE at 639 UI/mL (N: 0-100). Hepatotropic viruses and HIV were negative as well as syphilis screening, legionella, mycoplasma, leptospira, leishmaniosis, hydatids, fasciolosis, amebiasis and cysticercosis serologies. Myeloproliferative disease-associated mutation (JAK2 V617F) was not found.

Despite ongoing antibiotics, there was no clinical or analytical improvement. Given the history of asthma, fever, and sinusitis symptoms refractory to antibiotic treatment, very elevated ESR and marked eosinophilia, an additional autoimmune study was performed and revealed strong positive ANCA-p anti-MPO antibodies (83 IU/mL; N < 3.5).

**Figure 2:**
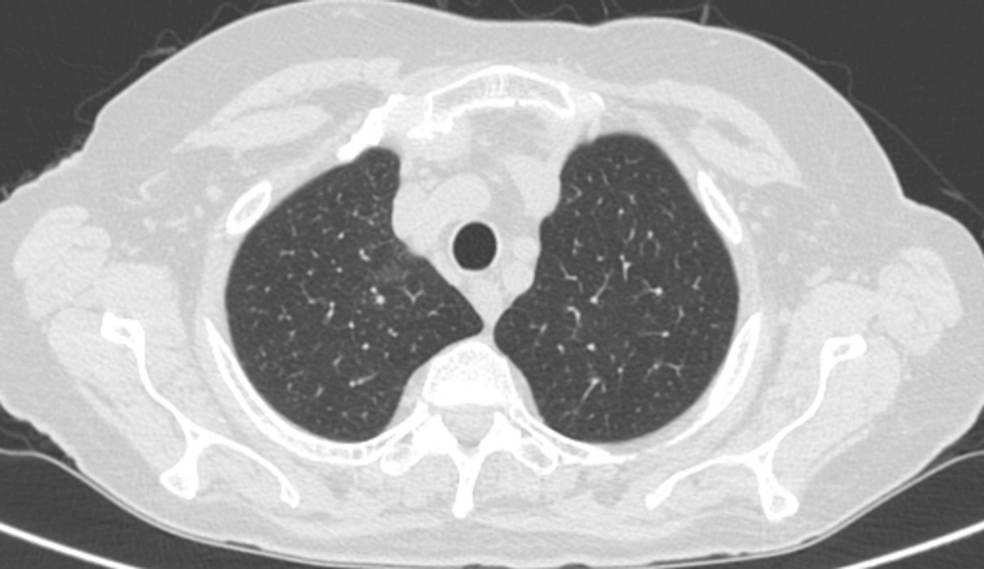
High-resolution chest CT showing diffuse centrilobular micronodules.

**Figure 3:**
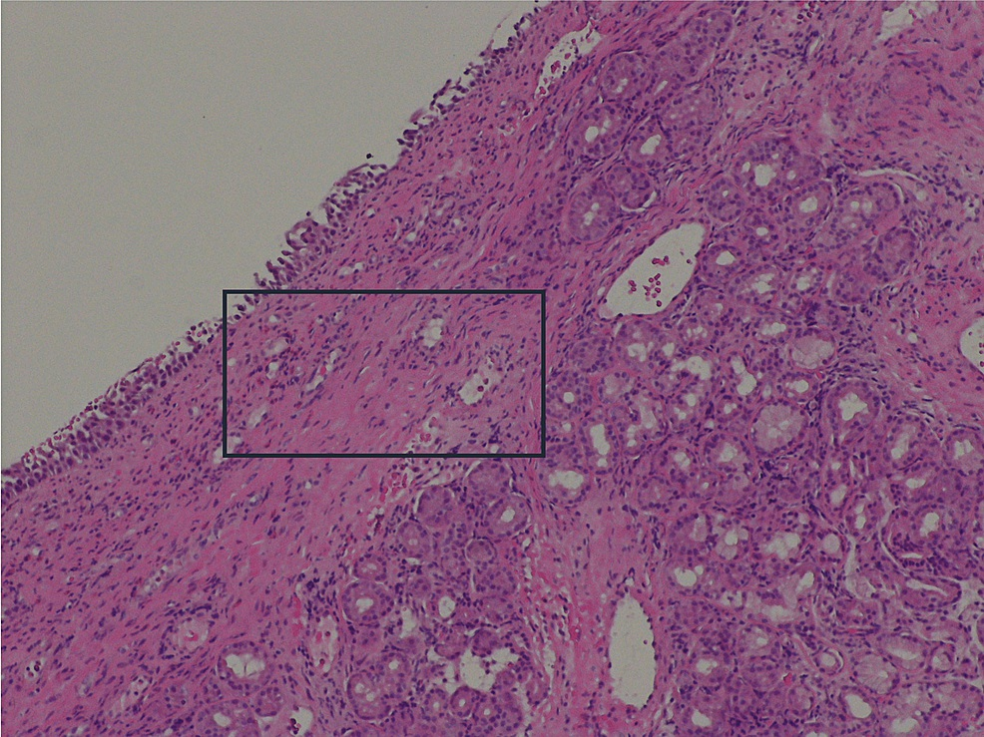
Eosinophils infiltration in the lamina propria and occupying blood vessels lumina (square) (HE, x200).

Furthermore, an extensive workup to evaluate expected organ involvement was performed with a full-body CT scan revealing lung, mesenteric and ganglionary involvement (Figure 2). Electromyogram was normal. Due to a high suspicion of EGPA, the patient was submitted to a biopsy of the paranasal sinuses, antibiotics were suspended, and she was started on corticosteroid (prednisolone 1 mg/kg/day). The sinuses biopsy later revealed tissue infiltration by eosinophils, confirming EGPA (Figure 3).

Symptoms improved gradually, despite corticosteroid tapering, until full resolution. At the six-month follow-up, there were no signs of active disease under prednisolone 10 mg/day and azathioprine 50 mg/day.

## Discussion

EGPA, known as Churg-Strauss syndrome since its description in 1951 until 2012, is the rarest ANCA-associated vasculitis. It is defined as an eosinophilic necrotizing granulomatous inflammation, frequently involving the respiratory tract, with necrotizing vasculitis predominantly affecting small to medium vessels associated with eosinophilia and asthma. The prevalence ranges from 2 to 38 per million cases and has an incidence of 0.5 to 3.7 per million in European countries [1,6], with equal preponderance between genders. Mean age at diagnosis is 50 years [2]. Although its pathophysiology is not well understood, several factors that confer susceptibility to EGPA are described, such as environmental triggers (allergens, infections, drugs, etc.) and some immunogenic factors. Despite being classically considered as a Th2-mediated disease, there is evidence of involvement of Th1 and Th17 cells as well as antibodies and humoral response in its etiopathogenesis. EGPA is classified as a systemic vasculitis associated with the presence of ANCA, however, these are only identified in 40% of cases. Recently, elevated values of IgG4 have been observed in cases of EGPA with active disease, pointing to the possibility that EGPA belongs to the group of diseases related to IgG4. Nonetheless, eosinophilic infiltration and ANCA-induced endothelial damage are probably the most important mechanisms in the pathogenesis of EGPA [5].

EGPA is characterized by three stages with a variable duration which can overlap. The prodromal phase occurs usually with late onset of asthma, rhinitis, nasal polyposis, and rhinosinusitis. It is followed by the eosinophilic phase where peripheral and tissue eosinophilia in several organs is the hallmark. Lastly, the vasculitic phase is characterized by systemic vasculitis affecting peripheral nerves, heart, lung, gastrointestinal tract and kidney and associated with constitutional symptoms like fever, malaise, and weight loss [4,7,8].

Lungs are the most frequently affected organ in EGPA. All patients have asthma at diagnosis, with clinical features similar to common allergic asthma, however, they show late onset, around the fourth and fifth decades of life [9], preceding systemic manifestations in about 12 years. Our patient was diagnosed with asthma 11 years before and had normal spirometry, in line with only 30% of EGPA patients presenting fixed bronchial obstruction as described in the literature [10]. Two-thirds of the patients exhibit involvement of the lung parenchyma and although migratory pulmonary infiltrates are the most typical finding in EGPA, there is no pulmonary pattern in chest CT specific to the disease [5].

Ear, nose and throat (ENT) manifestations are frequent in EGPA, and frequently associated with asthma; chronic sinusitis, polyposis and paranasal sinus involvement have been described with incidences of 60-80%, 40% and 75% cases, respectively. ENT manifestations are more frequent in ANCA-positive patients [9]. About 30-40% of EGPA patients show diffuse lymphadenopathy, predominantly axillary and cervical lymph nodes [11].

Cardiac involvement develops in less than 50% of patients but represents the main cause of early death and the worst prognosis. The most common condition is endomyocardial infiltration by eosinophils, but arrhythmia, pericarditis and valvular disease can occur. Gastrointestinal involvement occurs secondary to eosinophilic mucosa infiltration predominantly affecting the small bowel, presenting with abdominal pain and hemorrhage. Peripheral neuropathy (70%), renal involvement (25%) and skin manifestations are characteristics of the vasculitic phase, associated with constitutional symptoms, like fever, weight loss and fatigue, sometimes with paradoxical improvement of asthma symptoms. The typical presentation of peripheral neuropathy is multiplex mononeuritis or mixed sensorimotor peripheral neuropathy; central nervous system involvement occurs in 25% of cases. Renal manifestations are less common, and the typical presentation is pauci-immune focal and segmental necrotizing glomerulonephritis. Skin involvement is frequent, and purpura and nodules on the limbs and scalp are the most frequent manifestations [5].

The main differential diagnoses of EGPA are hypereosinophilic syndrome (HES), drugs hypersensitivity reactions, parasitic infections and other vasculitis such as granulomatosis with polyangiitis (GPA) and microscopic polyangiitis (MPA). Bronchopulmonary allergic aspergillosis, and acute and chronic eosinophilic pneumonia should also be excluded [4].

Marked peripheral eosinophilia (usually >1500 cells/uL or >10%) characterizes active EGPA, often associated with increased erythrocyte sedimentation rate. Normocytic normochromic anemia and elevated serum immunoglobulin E (IgE) can be present [4,7,8]. ANCA, while not specific and not always positive in EGPA, may be one effective method of investigation when present and may arise years before the vasculitis phase. P-ANCA positivity must be confirmed by the presence of myeloperoxidase in serum (anti-MPO) [2]. CT, electromyogram and echocardiogram and urine analysis are useful to evaluate common organ involvement. Bronchoalveolar lavage should be done in the presence of marked respiratory symptoms and/or relevant changes in chest CT [1,4].

Histological tissue biopsy of an affected organ is the most effective method of investigation. Eosinophil tissue infiltration and necrotizing granulomas are the hallmark histological findings in EGPA, however, the absence of granulomas in biopsy is not uncommon [1,7]. Perinasal sinus biopsy is limited by the low diagnostic profitability rate, with the observation of vasculitis or eosinophilic granuloma in less than 10% of nasal mucosa biopsies, reaching 50% in deep nasal sinus biopsy [9].

Our patient had positive p-ANCA (anti-MPO), marked eosinophilia and high IgE, as an elevation of inflammatory markers. Paranasal sinus biopsy revealed tissue infiltration by eosinophils. We confirmed EGPA diagnosis applying American College of Rheumatology (ACR) criteria (requires the presence of four or more criteria). In this clinical case, four criteria were met: asthma, eosinophilia >10%, paranasal sinusitis and extravascular eosinophil infiltration on biopsy findings. Other criteria are neuropathy, mononeuropathy or polyneuropathy and pulmonary infiltrates [12].

EGPA treatment depends on the severity of the disease. The five factors score (FFS) may be a tool to evaluate prognosis (gastrointestinal involvement, CNS involvement, cardiac involvement, proteinuria >1 g/24 h and serum creatinine ≥141 umol/L - each item assigns one point). Patients without poor prognosis (FFS=0) should be treated with corticosteroids alone (1 mg/kg/day of prednisone); additional treatment with immunosuppressants (azathioprine, cyclophosphamide, methotrexate) may be required in treatment failure, relapses or corticosteroids sparing. In severe disease, methylprednisolone pulses (15 mg/kg for three days) may be added. In patients with FFS ≥ 1, it has been recommended addition of cyclophosphamide pulses to corticosteroid treatment [4,13]. New management guidelines of ACR conditionally recommend initiating treatment with mepolizumab and glucocorticoids or methotrexate/azathioprine/mycophenolate mofetil and glucocorticoids over glucocorticoids alone on active non-severe disease, but with very low to low level of evidence. Rituximab is a recommended therapy in severe or non-severe acute EGPA [14]. Our patient started prednisolone therapy (1 mg/kg/day) with tapering after symptoms resolution. Azathioprine was later added to spare corticosteroids and prevent their known adverse effects. Remission is assumed when a prednisone dose of 7.5 mg/day is used to control systemic manifestations [1].

Three-month mortality reaches 50% in untreated EGPA patients. The introduction of corticosteroid therapy has significantly improved prognosis, with 5- and 10-year survival rates of 88-97% and 78-89%, respectively [2,8]. ENT involvement is associated with a better survival rate [8,9]. Nonetheless, about half of the EGPA patients relapse at five years after disease onset [2]. In only a minority of patients, it is possible to suspend corticosteroid therapy, essential to reach the lowest possible corticosteroid dose to control the disease and prevent adverse effects [4]. Asthma exacerbations and/or ENT manifestations are frequent, multifactorial, and should not systematically be considered EGPA relapse [1,4].

## Conclusions

Diagnosis is a challenge, and clinical clues are very important. This case shows that EGPA should be considered in the presence of late-onset asthma, sinusitis, and eosinophilia, especially when standard asthma/sinusitis treatment is not improving the patient's condition and the complaints persist in time. Early diagnosis allows treatment simplification, delaying complications and mortality reduction.
